# Quality control and signaling pathways at stalled ribosomes

**DOI:** 10.1038/s12276-025-01623-w

**Published:** 2026-01-15

**Authors:** Weili Denyse Chang, Young-Jun Choe

**Affiliations:** School of Biological Sciences, Nanyang Technological University, Singapore, Singapore

**Keywords:** Ribosome, Protein aggregation, Protein quality control, Ubiquitylation, Endoplasmic reticulum

## Abstract

Aberrant mRNAs can arise from errors in RNA processing or from various physicochemical insults. Ribosomes translating such faulty mRNAs may stall, producing incomplete and potentially toxic polypeptides. These aberrant translation products are eliminated by the ribosome-associated quality control pathway. Ribosome stalling also leads to ribosome collisions, which can activate signaling pathways that enable cells to adapt to stress or determine cell fate. Here, in this Review, we summarize the molecular mechanisms of ribosome stalling and the associated quality control and signaling pathways, and discuss their implications in disease and therapeutics.

## Ribosome stalling mechanisms and their impact on cell health

Proteins sometimes fold incorrectly, which can cause problems in cells. This Review explores how ribosomes can stall and lead to protein misfolding. The researchers review how ribosome stalling happens and how cells respond, focusing on a process called ribosome-associated quality control (RQC). RQC helps degrade faulty proteins and is crucial for maintaining cell health. The study explains that ribosome stalling can occur due to damaged RNA or specific sequences in the genetic code. Cells have mechanisms to resolve these stalls, such as splitting the ribosome into parts and degrading the faulty protein. Research highlights that defects in these processes are linked to aging and diseases such as neurodegeneration. The researchers conclude that understanding RQC better could lead to new treatments for diseases caused by protein misfolding. Future research may uncover more about how cells manage protein production and maintain health.

This summary was initially drafted using artificial intelligence, then revised and fact-checked by the author.

## Introduction

**Fig. 1:**
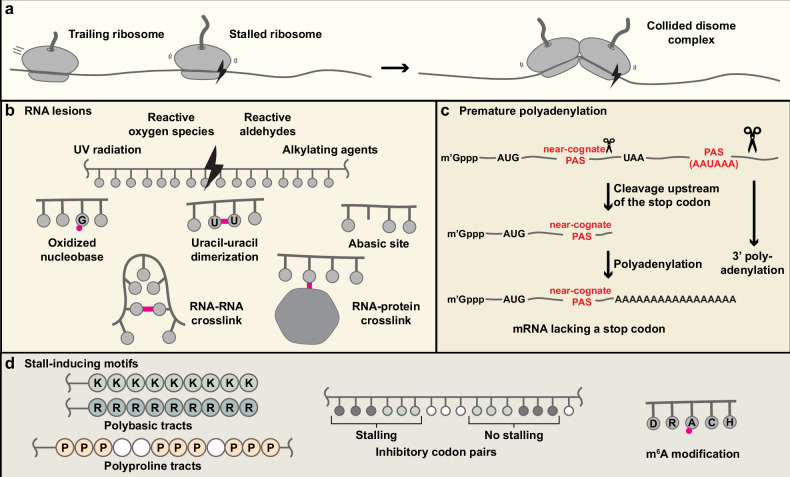
Ribosomes can stall during translation elongation. **a** Stalled ribosomes collide with trailing ribosomes, forming disome complexes. **b** RNA damage caused by chemical or physical stress can induce stalling. **c** mRNAs lacking a stop codon can arise when a near-cognate polyadenylation signal (PAS) is present within the coding sequence. The canonical PAS motif is AAUAAA. The 5′-cap is also shown (m^7^Gppp). **d** Specific RNA sequences or nascent peptide features can inherently cause ribosome stalling. m^6^A modification typically occurs within the consensus sequence DRACH (D = A/G/U, R = A/G, H = A/C/U).

Misfolded proteins not only lose function but can also form harmful aggregates that disrupt protein homeostasis (proteostasis) by sequestering protein quality control factors^1^. One potential source of misfolded proteins is incomplete nascent polypeptides generated by prematurely stalled ribosomes. Elongating ribosomes can stall upon encountering RNA damage or specific sequence motifs. If not promptly resolved, trailing ribosomes collide with stalled ribosomes, forming di-ribosome (disome) complexes^2–4^ (Fig. 1a). Collided ribosomes act as markers of translational stress and recruit factors that initiate rescue, quality control and stress response pathways. This Review summarizes the causes of ribosome stalling and cellular responses to ribosome collisions, with a focus on ribosome-associated quality control (RQC), which directs stalled polypeptides to proteasomal degradation^5,6^. RQC is critical for maintaining cellular proteostasis^7–9^, and defects in this pathway have been implicated in aging^10,11^ and a range of diseases^12–16^. A deeper understanding of RQC and other molecular events at stalled ribosomes may ultimately inform the development of new therapeutic strategies.

## Causes of ribosome stalling

**Fig. 2:**
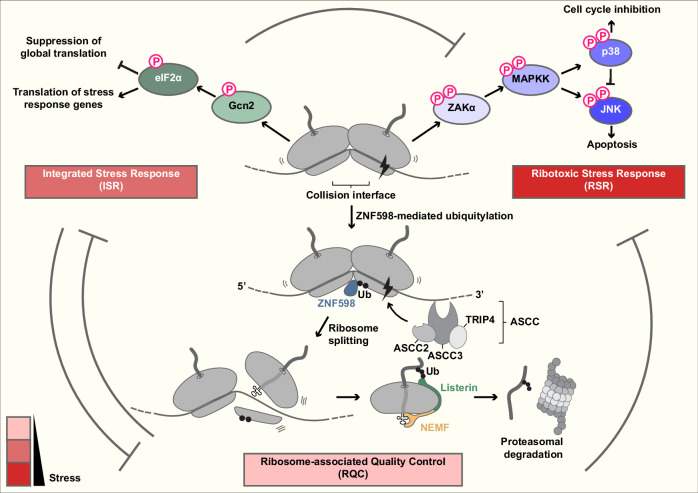
Collided ribosomes can activate three major response pathways. The disome interface is recognized by the E3 ligase ZNF598, which ubiquitylates 40S proteins such as eS10 and uS10. This modification recruits the ASCC to mediate ribosome splitting. The resulting 60S subunit, still attached to the stalled nascent polypeptide, is then processed by the RQC pathway, which targets the stalled polypeptide for proteasomal degradation. Persistent stalling stress activates broader cellular responses, including the ISR and RSR. These signaling cascades help mitigate stalling and influence cell fate decisions. The RQC, ISR and RSR pathways exhibit cross-talk, with activation of one often suppressing the others. The dominant pathway is probably determined by the severity and duration of ribosome stalling. Ub, ubiquitin; P, phosphate.

Ribosome stalling during translation elongation can be broadly attributed to two causes: (1) RNA lesions resulting from physicochemical insults (Fig. 1b) and (2) inherent stall-inducing motifs within coding sequences (Fig. 1c,d). Defective mRNAs are typically detected during translation; thus, at least one round of aberrant protein synthesis is often unavoidable^17^.

## RNA lesions

Because of their single-stranded nature and lack of higher-order packing, mRNAs are particularly susceptible to damage^18,19^. Their cytosolic localization exposes them to reactive molecules generated during endogenous metabolism, as well as to environmental toxins taken up by the cell (Fig. 1b).

The mitochondria are a major source of cellular reactive species, producing superoxide as a by-product of oxidative metabolism^20^. Superoxide is subsequently converted into other reactive oxygen species, such as hydrogen peroxide (H₂O₂) and hydroxyl radicals (HO•)^20^. Although reactive oxygen species serve essential functions in redox signaling^21^, their high reactivity also damages cellular components, including nucleic acids^22^. Oxidative reactions can produce modified nucleobases such as 8-oxo-7,8-dihydroguanine, which induce ribosome stalling by disrupting codon–anticodon pairing^23^. Oxidative damage can also create abasic sites and cause strand breaks^24^. Notably, RNA oxidation has been associated with neurodegenerative diseases^25^.

Aldehydes damage nucleic acids by forming covalent protein–RNA crosslinks, which physically obstruct ribosome translocation^26,27^. Formaldehyde is produced endogenously during one-carbon metabolism and demethylation reactions, or introduced through exposure to environmental toxins such as tobacco smoke^28^. In the liver, acetaldehyde is generated as a toxic intermediate during alcohol metabolism^29^. Alkylating agents, another reactive species, covalently modify nucleobases by adding alkyl groups, such as methyl groups, to oxygen or nitrogen atoms^22^. These agents can originate from both endogenous and exogenous sources, including metabolic by-products of lipid peroxidation and nitrosamines present in tobacco smoke^30^. Alkylated bases disrupt canonical Watson–Crick base-pairing interactions, thereby inducing ribosome stalling^22,31^. Finally, ultraviolet (UV) radiation is a physical source of nucleic acid damage, inducing dimerization of adjacent uracil nucleobases and the formation of RNA–RNA or RNA–protein crosslinks^32^. These RNA photolesions physically obstruct ribosome elongation during translation and have been shown to activate cellular pathways that respond to ribosome stalling^33,34^.

## Stall-inducing motifs

Even in the absence of external insults, aberrant mRNAs that trigger ribosome stalling can arise from errors in mRNA processing. In addition, specific sequence motifs within intact transcripts can intrinsically slow elongation. Such pauses may result from inefficient A-site decoding, unfavorable peptide bond formation or nascent polypeptides that impede ribosomal function.

One prominent stall-inducing motif is the polyadenosine (poly(A)) tract. The polyadenylation signal (PAS) in pre-mRNAs triggers downstream cleavage and 3′ poly(A) extension^35^ (Fig. 1c). Poly(A) is critical for transcript stability and promotes translation efficiency^36^. Because translation terminates at stop codons, ribosomes do not reach the downstream poly(A) tail. However, if near-cognate PAS elements occur upstream of a stop codon, premature polyadenylation can generate mRNAs lacking a stop codon^37^ (Fig. 1c). Consequently, ribosomes translate into the poly(A) tail, producing polylysine tracts that stall elongation^38^.

Early in vitro translation studies using rabbit reticulocyte lysates showed that polybasic sequences such as polylysine and polyarginine slow translation through electrostatic interactions with the negatively charged ribosomal exit tunnel^39^. Subsequent cell-based studies found that stalling is stronger on polylysine tracts encoded by consecutive AAA codons than on equivalent tracts encoded by the alternative lysine codon AAG^40,41^, suggesting that the mRNA sequence also contributes to stalling. Later studies revealed that poly(A) mRNA adopts a helical conformation that hinders codon–anticodon pairing at the A-site, thereby inducing stalling^42,43^.

Other polyamino acid tracts can also slow elongation. Proline is a poor donor and acceptor in peptide bond formation due to its bulky cyclic side chain and imino character, which can cause ribosome stalling^44,45^. However, the universally conserved translation elongation factor eIF5A facilitates translation through consecutive proline residues^46^. Polytryptophan tracts have also been shown to induce ribosome stalling^47^. Although the precise molecular mechanism remains to be fully elucidated, the bulkiness of tryptophan side chains probably clogs the ribosomal exit tunnel^47^.

Codon usage is another key determinant of translation elongation rate. In general, optimal codons are decoded more rapidly than rare codons^48^. In *Saccharomyces cerevisiae*, repeats of the rare CGA codon have been widely used to induce ribosome stalling^49^. However, CGA-induced stalling is not solely attributable to codon rarity. Notably, the identity of the P-site tRNA can influence decoding efficiency at the A-site^50^; thus, effective translation inhibition requires two adjacent CGA codons occupying the P- and A-sites simultaneously. This neighboring codon effect is not limited to the CGA–CGA dicodon. For example, the CUC–CCG dicodon also reduces translation efficiency, suggesting that the cognate tRNA for the CUC codon in the P-site interferes with decoding of the CCG codon in the A-site^50^. By contrast, the reverse dicodon, CCG–CUC, does not reduce translation efficiency^50^ (Fig. 1d; inhibitory codon pairs), underscoring the importance of codon context (that is, sequence) in ribosome stalling rather than codon optimality alone. In yeast, 17 inhibitory codon pairs have been identified^50^. Notably, many of these include CGA and CUG codons, which are decoded via wobble base pairing: I·A for CGA and U·G for CUG.

Inosine, as in the I·A pair, is a common modification at the wobble position of tRNAs (nucleotide 34, the first base of the anticodon) arising from deamination of adenosine^51^. Although nucleotide modifications are most prevalent in tRNAs and rRNAs, mRNAs also carry several chemical marks, among which *N*^6^-methyladenosine (m^6^A) is the most abundant^52^ (Fig. 1d; m^6^A modification). m^6^A is deposited by a nuclear methyltransferase complex comprising METTL3 and METTL14 subunits, which methylates adenosine at the *N*^6^ position^53^. Although m^6^A-modified codons can still base pair with U in the anticodon, their decoding is slowed^54^, increasing ribosome collisions^55^.

## Cellular responses to ribosome stalling

When ribosomes stall and collide, cells activate downstream pathways with two main functions: (1) resolving stalled ribosomes by splitting and recycling subunits, a process preceding the RQC pathway; and (2) preventing additional stalling events. If stalling persists, cells further engage broader stress responses, including the integrated stress response (ISR) and the ribotoxic stress response (RSR).

## Resolution of stalled ribosomes

Canonical translation termination at stop codons is mediated by eukaryotic release factor 1 (eRF1), delivered to the ribosome by the translational GTPase eRF3^56^. Upon eRF1 accommodation at the A-site, its conserved GGQ (Gly–Gly–Gln) motif catalyzes peptidyl-tRNA hydrolysis, releasing the polypeptide^57^. Termination is followed by ribosome recycling, during which the ATPase ABCE1 (Rli1 in yeast) dissociates the 80S ribosome into 60S and 40S subunits^58,59^. Because eRF1 specifically recognizes stop codons, it cannot resolve stalled ribosomes. Instead, a homologous protein, Pelota in metazoans and Dom34 in yeast, is delivered by the GTPase Hbs1 (an eRF3 homolog) to the empty A-site of ribosomes stalled at the 3′ end of truncated mRNAs^60,61^. As in canonical termination, the ATPase ABCE1/Rli1 mediates ribosome splitting^62^. However, unlike eRF1, Pelota/Dom34 lacks the GGQ motif required for peptidyl-tRNA hydrolysis, leaving the peptidyl-tRNA attached to the large ribosomal subunit.

If ribosomes stall within a coding sequence rather than at the 3′ end, the A-site may not be readily accessible to Pelota/Dom34. In such cases, ribosome collisions activate an alternative splitting mechanism. The unique 40S–40S interface formed between collided ribosomes is recognized by an E3 ubiquitin ligase^3,4^ (Fig. 2). In mammals, ZNF598 ubiquitylates eS10 (K138, K139) and uS10 (K4, K8)^63,64^, whereas in yeast, Hel2 targets uS10 at K6 and K8^65^. While disomes (two collided ribosomes) represent the minimal substrate for ZNF598/Hel2 recognition, trisomes (three collided ribosomes) are targeted more efficiently for ubiquitylation^66^.

Ubiquitylated 40S ribosomal proteins in disomes or trisomes recruit the ribosome-splitting machinery ASC-1 complex (ASCC) in mammals or RQC-trigger (RQT) complex in yeast^65–67^. The ASCC/RQT complex comprises the RNA helicase ASCC3/Slh1, the ubiquitin-binding protein ASCC2/Cue3 and the zinc-finger protein TRIP4/Rqt4. Cue3 and Rqt4 bind Hel2-ubiquitylated ribosomal proteins^68^. Slh1 engages the 3′ mRNA overhang extending from the lead, stalled ribosome and exerts a pulling force on the transcript, powered by ATP hydrolysis. This action pulls the trailing ribosome forward, generating the force to split the stalled ribosome positioned between the RQT complex and the trailing ribosome^69^. Similar to Dom34-mediated splitting, the large ribosomal subunit split by the ASCC/RQT complex retains peptidyl-tRNA, which is subsequently degraded (Fig. 2; see also ‘RQC’ section). The ZNF598/Hel2-ubiquitylated small subunit is recycled by deubiquitinases: USP10 in mammals^70^ and Ubp2 and Ubp3 in yeast^71,72^.

## Cellular mechanisms to minimize ribosome stalling

Even after stalled ribosomes are resolved, defective mRNAs may continue to be translated, causing recurrent stalling. To prevent this, cells activate additional collision-responsive pathways that suppress further stalling and limit the accumulation of aberrant translation products.

In human cells, EDF1 functions as a collision sensor that acts independently of ZNF598, recruiting the translational repressors GIGYF2 and 4EHP (eIF4E-homologous protein) to collided ribosomes^73–75^. The locally enriched 4EHP then outcompetes eIF4E for binding to the 5′ cap of the stall-inducing mRNA, thereby blocking translation initiation because 4EHP cannot interact with eIF4G^76,77^. This mechanism directly prevents further translation of the target mRNA, complementing ZNF598-mediated ribosome rescue. Because EDF1 is far more abundant than ZNF598, it may function as an upstream collision sensor^74,75^. However, ZNF598 activity does not depend on EDF1^74,75^.

Stalled ribosomes also trigger mRNA decay pathways, including no-go decay and nonstop decay, which irreversibly eliminate faulty transcripts and thereby prevent further production of aberrant proteins. The mechanisms underlying these pathways have been reviewed elsewhere^78^. Notably, ribosome-induced mRNA decay not only safeguards against defective transcripts but may also regulate gene expression. For instance, m^6^A modification can destabilize mRNAs through a decay process mediated by YTH domain-containing family proteins (YTHDF)^79,80^. Notably, m^6^A marks within coding regions promote mRNA decay more strongly than those in untranslated regions^81,82^. As described earlier, m^6^A slows translation and induces ribosome collisions^54,55^. The collided ribosomes recruit YTHDF, enhancing their binding to m^6^A and thereby promoting mRNA decay under normal conditions^55^. Under stress conditions, however, global translation is reduced by the ISR (discussed in the following section), resulting in fewer ribosome collisions and, consequently, greater stability of m^6^A-modified mRNAs^55^. This mechanism may explain the increased abundance of certain m^6^A-modified transcripts during stress^83,84^. m^6^A-dependent mRNA decay can also alter transcript profiles in cancer cells. Translation of codons bearing m^6^A at the wobble position (the third nucleotide of the codon) is facilitated by a corresponding modification in the cognate tRNAs, in which U at the first anticodon position is modified to mcm^5^s^2^U (ref. ^82^). Thus, the balance between m^6^A and mcm^5^s^2^U modifications can determine mRNA stability. When mcm^5^s^2^U modification is insufficient, ribosomes stall and collide at m^6^A-modified codons, promoting mRNA decay^82^. Interestingly, cancer cells tend to reduce m^6^A levels while enhancing mcm^5^s^2^U modification, thereby stabilizing transcripts involved in oncogenic signaling pathways^82^.

When ribosome stalling arises from mRNA–protein crosslinks or from elongation or termination factors trapped within the ribosome, degradation of the stall-inducing proteins is promoted^26,27,85,86^. This pathway involves the additional collision sensor, GCN1, which associates with collided ribosomes and the E3 ubiquitin ligases RNF14 and RNF25. RNF25-mediated ubiquitylation of the small ribosomal subunit protein eS31 facilitates RNF14-driven polyubiquitylation of stall-inducing proteins, targeting them for proteasomal degradation. Notably, the RQC pathway is activated independently of the GCN1–RNF25–RNF14 axis^26^^,^^27^, underscoring how multiple quality control mechanisms cooperate to resolve ribosome stalling stress.

## Stress response pathways

Cellular sensing of ribosome collisions can also activate stress response pathways. These include the ISR and the RSR (Fig. 2), which protect cells from persistent translational stress and influence cell-fate decisions.

The ISR is regulated by four upstream kinases, each activated by distinct stress signals^87,88^. One of these kinases, GCN2, was originally identified for its role in nutrient sensing^89^. During amino acid starvation, elevated levels of uncharged tRNAs activate yeast GCN2 through their direct binding to the histidyl-tRNA synthetase-like domain of GCN2^90,91^. Activated GCN2 phosphorylates the α subunit of eIF2 (eIF2α)^92^. eIF2 forms a ternary complex with Met-tRNA_i_^Met^ and GTP, which, together with other initiation factors, assembles onto the 40S subunit to form the 43S pre-initiation complex (PIC)^93^. As the PIC scans the 5′ untranslated region of the mRNA, the start codon is recognized through codon–anticodon pairing with Met-tRNA_i_^Met^, delivered by eIF2^93^. This recognition triggers GTP hydrolysis by eIF2, leading to the dissociation of initiation factors and joining of the large ribosome subunit^93^. eIF2 bound to GDP is normally recycled to its active GTP-bound form by the guanine nucleotide exchange factor eIF2B, permitting subsequent rounds of translation initiation^93^. However, phosphorylated eIF2α inhibits eIF2B^94,95^, leading to a gradual depletion of functional eIF2-GTP and impairing PIC formation. As a result, the ISR suppresses global translation.

Notably, recent studies have identified collided ribosomes—rather than uncharged tRNAs—as critical activators of the ISR kinase GCN2 (Fig. 2). Early work using mice carrying mutations in a brain-specific tRNA (tRNA^Arg(UCU)^) and in the putative ribosome recycling factor GTPBP2 revealed that ribosomal stalling can activate GCN2 independently of uncharged tRNA accumulation^96^. Subsequent studies showed that the ribosomal P-stalk is required in vivo and sufficient in vitro for GCN2 activation^97,98^, establishing a direct molecular link between the ribosome and ISR signaling. These findings support a model in which the P-stalk of stalled ribosomes activates GCN2. In actively translating ribosomes, however, the P-stalk, part of the GTPase-associated center, would be occupied by elongation factors (eEF1A and eEF2), preventing its interaction with GCN2^97^^,^^98^. Importantly, titration of the ribosome inhibitor anisomycin revealed that GCN2 is activated by collided ribosomes rather than by stalled ribosomes themselves^33^. Intermediate concentrations of anisomycin, which induce ribosome collisions, activated GCN2, whereas higher concentrations that cause more severe stalling and thus fewer collisions failed to do so^33^. GCN1 has long been recognized as a critical co-activator of GCN2^99^. Consistent with the notion that ribosome collisions act as an ISR trigger, cryo-electron microscopy analyses revealed that GCN1 associates with disomes^100^. Supporting this model, GCN1-selective ribosome profiling demonstrated enrichment of disome footprints^101^. More recently, Mbf1 (the yeast homolog of EDF1), which also binds collided ribosomes, was identified as an additional co-activator of Gcn2^102^. Interestingly, under normal conditions, yeast GCN2 associates with the nontranslating free 60S subunit as a homodimer^103^. Taken together, these findings suggest that GCN2, positioned in a stand-by state on the 60S subunit, can be redistributed to colliding ribosomes through its interaction with the critical co-activator GCN1. During activation, homodimeric GCN2 undergoes structural rearrangement and autophosphorylation^104^. Although uncharged tRNAs alone may not be sufficient to activate GCN2 without ribosome collisions, their accommodation at the A-site of stalled ribosomes can further enhance GCN2 activation^105^. The mechanistic details of GCN2 activation may differ depending on how ribosome collisions are induced^106^.

The RSR is another cellular pathway activated by ribosome stalling and collisions^33,107^ (Fig. 2). Its activation triggers a mitogen-activated protein kinase (MAPK) cascade that ultimately results in the activation of downstream MAPKs, including p38 and c-Jun N-terminal kinase (JNK). These kinases elicit cellular outcomes ranging from restoration of homeostasis to cell death, which may depend on the severity of stress^33,34,108^. ZAK, a MAPKKK, functions as the upstream activator of the RSR. Specifically, ZAKα, the longer of the two major ZAK isoforms, α and β, binds to ribosomes through its flexible C-terminal domain, where it is activated via autophosphorylation^107,108^. The kinase then dissociates from ribosomes and mediates downstream signaling^107,108^. At present, it remains unclear whether ZAKα specifically binds collided ribosome complexes. Mild ribosome stalling drives ZAKα-mediated p38 activation, which temporarily restricts cell cycle progression and regulates factors that resolve stalled ribosomes and prevent further stalling, thereby promoting recovery from translational stress^34,108^. By contrast, when cells experience severe stalling stress, for instance from extensive UV-induced mRNA damage, ZAKα-dependent activation of JNK can promote cell death^33,108^. Although UV radiation also activates the DNA damage response, the RSR—not the DNA damage response—primarily mediates early apoptosis in this context^108^.

Collided ribosomes may influence a broader spectrum of signaling pathways beyond the ISR and RSR, not necessarily through direct activation of protein kinases. One prominent example is the cGAS–STING pathway of the innate immune response. Upon viral infection, cytosolic foreign DNA is detected by cyclic GMP–AMP synthase (cGAS)^109^. Activated cGAS synthesizes the second messenger cyclic GMP–AMP^110^, a cyclic dinucleotide that binds to the cytosolic ligand-binding domain of the endoplasmic reticulum (ER) transmembrane protein STING (stimulator of interferon genes)^111^. Upon ligand binding, STING recruits TANK-binding kinase 1 (TBK1) and interferon regulatory factor 3 (IRF3), enabling TBK1-mediated phosphorylation and activation of IRF3^112,113^. The activated transcription factor IRF3 then drives the expression of type I interferons, which in turn suppress viral replication. Notably, collided ribosomes can interact with cGAS, enhancing its DNA-dependent synthesis of cyclic GMP–AMP^114^. During viral infection, the host translation system may experience increased ribosome collisions due to the heavy burden of viral protein production, which often relies on atypical translation mechanisms such as frameshifting and stop-codon readthrough^115^. Overall, ribosome collisions potentiate the cGAS–STING pathway by stimulating production of the second messenger.

## Cross-talk between signaling pathways at stalled ribosomes

Ribosome collisions can activate three major pathways: the ISR, the RSR and the RQC (Fig. 2). The severity of collisions, including their frequency and duration, probably determines which pathway is engaged. Once ZNF598/Hel2 directs collided ribosomes into the RQC pathway, activation of the ISR and RSR is prevented because the signaling platform—the disome—is dismantled. For example, Hel2 has been shown to inhibit Gcn2-mediated phosphorylation of eIF2α^116,117^. Similarly, ZNF598 attenuates activation of the RSR pathway^33^. Consistent with this antagonistic relationship, activation of the ISR also suppresses both the RQC^116^ and RSR pathways^33^. By reducing global translation, the ISR lowers the frequency of ribosome collisions, thereby limiting the activation of collision-dependent pathways. Under mild translational stress, both the RQC and ISR pathways promote cellular protection and the restoration of proteostasis. However, under severe or prolonged stress, RSR-mediated activation of JNK can shift the balance toward apoptosis^108^.

## RQC

RQC was first identified in *S. cerevisiae*^5,6^. It comprises several factors that act on the 60S ribosomal subunit, including Rqc2 (NEMF in mammals), the E3 RING ubiquitin ligase Ltn1 (Listerin/LTN1 in mammals), Rqc1 (TCF25 in mammals) and the AAA-ATPase Cdc48 (VCP in mammals). The 60S subunits that dissociate from stalled ribosomes retain a peptidyl-tRNA anchored at the P-site. The presence of this tRNA at the intersubunit interface is recognized by Rqc2/NEMF, which subsequently recruits Ltn1/LTN1 to the 60S subunit^118,119^ (Fig. 2). The C-terminal RING domain of Ltn1 is positioned near the ribosomal exit tunnel, where it ubiquitylates stalled polypeptides^118,119^. Rqc1/TCF25 promotes the formation of K48-linked polyubiquitin chains during this process^120^. The polyubiquitylated nascent polypeptides are subsequently released from the 60S. All tRNAs possess a conserved CCA sequence at their 3′ end, which is added during tRNA maturation. The CCA tail of the peptidyl-tRNA anchored on the 60S subunit is cleaved by the endonuclease Vms1/ANKZF1^121^. Cdc48/VCP then extracts polyubiquitylated stalled polypeptides from 60S for proteasomal degradation^122^. In human cells, tRNAs with truncated 3′ ends are restored by the CCA-adding enzyme tRNA nucleotidyl transferase 1 (TRNT1), enabling their subsequent aminoacylation^123^.

## RQC and UFM1 at the ER

**Fig. 3:**
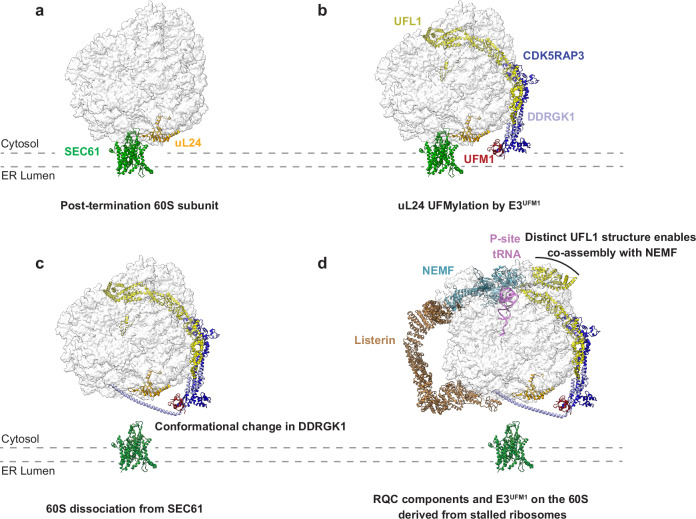
Detachment of the 60S subunit from the SEC61 translocon is facilitated by UFMylation. **a** A 60S ribosomal subunit remains associated with the ER translocon after completion of secretory-protein translation. Note that no peptidyl-tRNA remains attached. PDB ID: 8OJ0^133^. **b** The E3^UFM1^ complex associates with the 60S subunit bound to SEC61. UFM1 is conjugated to uL24, although the structure of this linkage has not been resolved. The UFL1 binding site overlaps with the 40S interface (see **d** for the position of the P-site), preventing UFMylation of 80S ribosomes. Note that the E3^UFM1^ remains bound to the 60S subunit after UFMylation. PDB ID: 8OJ0^133^. **c** UFMylation of uL24 induces a conformational change in DDRGK1 (which contains the long α-helix spanning the region between the ribosomal exit tunnel and SEC61), leading to detachment of the 60S subunit. PDB ID:8OJD^133^. **d** The UFL1 binding site at the intersubunit interface overlaps with that of NEMF (see **c** and **d**). The alternative conformation of UFL1 enables the E3^UFM1^ to co-assemble with NEMF on the same 60S subunit. PDB ID: 9GY4^134^. Note that the 60S subunit detached from SEC61 in **c** and **d** would remain tethered to the ER membrane, as DDRGK1 is a transmembrane protein. The structural details of DDRGK1 membrane insertion have not been resolved.

Secretory proteins are cotranslationally translocated into the ER. This process begins when the signal recognition particle binds to the signal sequence emerging from the ribosome and directs the translating ribosome to the ER membrane via interaction with the signal recognition particle receptor, an integral membrane protein complex^124^. The ribosome is then aligned with the SEC61 translocon, enabling nascent chains to be threaded directly into the ER lumen^125^. This tight coupling raises the question of whether the RQC can access and target stalled secretory polypeptides through the narrow gap between the ribosomal exit tunnel and the SEC61 translocon. Early studies demonstrated that stalled secretory polypeptides can be degraded by cytosolic RQC^126,127^. Subsequent work revealed that UFM1 conjugation (UFMylation) of the 60S ribosomal protein uL24 is essential for RQC at the ER^128,129^. UFM1 is a ubiquitin-like modifier whose conjugation requires an E3 ligase complex composed of three components: UFL1, DDRGK1 (also known as UFBP1) and CDK5RAP3^130^. DDRGK1, a transmembrane protein, anchors the UFM1 E3 ligase complex (E3^UFM1^) to the ER membrane, thereby restricting uL24 UFMylation to ribosomes at the ER rather than in the cytosol^128,129,131^. Because uL24 is positioned near the SEC61 translocon (Fig. 3a), its UFMylation has been proposed to weaken the interaction between 60S and SEC61 (Fig. 3b), thereby allowing the access of cytosolic RQC^128^. Subsequent studies revealed that a region of DDRGK1 within the E3^UFM1^ binds UFM1 conjugated to uL24 and occludes the contact site between the 60S subunit and SEC61, resulting in 60S detachment^132,133^ (Fig. 3c). Thus, the E3^UFM1^ functions not only as the writer but also as the reader of UFMylation. Cryo-electron microscopy structural analysis revealed that the E3^UFM1^ associates with 60S subunits lacking peptidyl-tRNA^132,133^ (Fig. 3b,c), suggesting that 60S subunits derived from canonical eRF1-dependent termination at stop codons are UFMylated to promote efficient 60S recycling after completion of secretory protein synthesis. UFMylation is prevented during elongation because the 40S subunit masks the intersubunit interface of the 60S, which is thought to be the key site for the initial binding of UFL1^133^, a subunit of the E3^UFM1^.

The elongated structure of the E3^UFM1^ wraps around the 60S subunit, spanning from the intersubunit interface to the exit tunnel^132,133^ (Fig. 3b). Notably, its binding site at the intersubunit interface overlaps with that of the RQC component NEMF, leaving it unclear how UFMylation and RQC are coordinated at the ER. A recent study showed that the UFL1 subunit in the E3^UFM1^ undergoes conformational adjustments in the presence of peptidyl-tRNA and NEMF^134^ (Fig. 3d). This structural flexibility allows the E3^UFM1^ and the RQC machinery to co-assemble on the same 60S subunit. Listerin can access stalled polypeptides once UFMylation displaces the 60S subunit from the SEC61 translocon. However, the detached 60S subunits—whether derived from canonical termination (Fig. 3c) or ribosome stalling (Fig. 3d)—remain associated with the E3^UFM1^. Subsequent deUFMylation of uL24 by UFSP2 ultimately recycles the 60S for reuse^135^. Failure of RQC at the SEC61 translocon not only disrupts ER homeostasis but may also impair global protein secretion by clogging the translocon^136^. Similarly, unresolved ribosome stalling during the translation of mitochondrial proteins can obstruct the translocase of the outer membrane (TOM) complex, thereby inhibiting mitochondrial protein import^136^.

## CAT tail extension

**Fig. 4:**
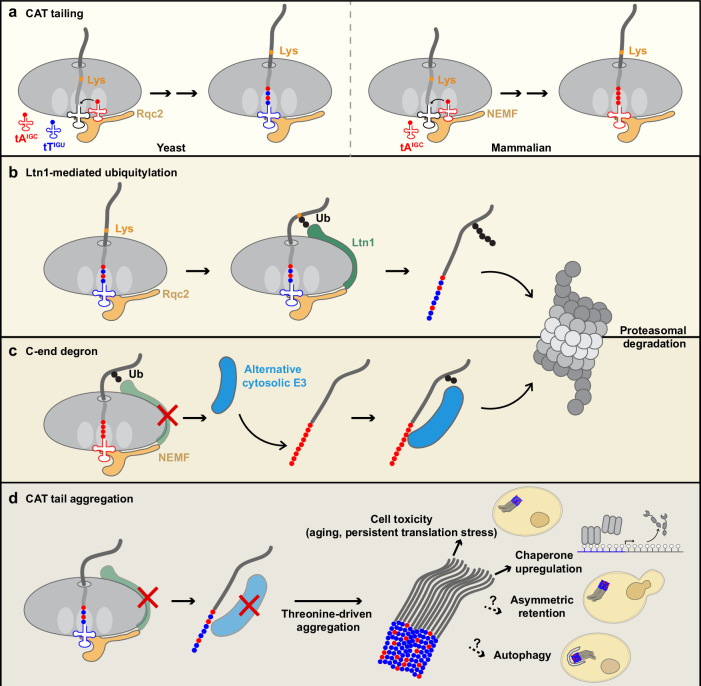
The effects of CAT tails. **a** Yeast Rqc2 and human NEMF extend the C-termini of stalled polypeptides on RQC-targeted 60S subunits without requiring an mRNA template. In yeast, Rqc2 specifically recruits tRNA^Ala(IGC)^ and tRNA^Thr(IGU)^, generating C-terminal Ala/Thr (CAT) tails. In humans, CAT tails consist predominantly of alanine. CAT tail elongation can expose previously buried lysine residues. **b** Ltn1 ubiquitylates lysine residues located near the ribosome exit tunnel. **c** If stalled polypeptides escape Ltn1-mediated ubiquitylation, their CAT tails can be recognized by alternative off-ribosome E3 ligases. **d** In yeast, undegraded CAT-tailed proteins form detergent-insoluble aggregates, driven by threonine residues. This aggregation may serve as a form of spatial quality control, benefiting cells under stress conditions, although chronic accumulation during aging can be toxic.

Although the positioning of the Ltn1 RING domain near the ribosomal exit tunnel is well suited for ubiquitylating stalled polypeptides^118,119^, this activity is limited when lysine residues are not readily accessible near the tunnel. Remarkably, Rqc2/NEMF overcomes this limitation by extending stalled polypeptides that remain anchored to the 60S subunit (Fig. 4a). Rqc2/NEMF recruits aminoacyl-tRNAs to the A-site of the 60S subunit, enabling peptide bond formation at the peptidyl transferase center^118,137,138^. Consequently, stalled polypeptides can be elongated on the 60S subunit via a noncanonical form of translation that does not require the 40S subunit and mRNA templates. Because the ribosomal exit tunnel accommodates approximately 30–40 amino acids, lysine residues may, by chance, be buried within it. C-terminal elongation of stalled polypeptides can expose these hidden lysines beyond the tunnel, rendering them accessible for ubiquitylation by Ltn1^139^ (Fig. 4b). This C-terminal extension of stalled polypeptides was first identified in yeast, where Rqc2 selectively recruits alanyl- and threonyl-tRNAs^118^. Thus, the extension was termed the C-terminal Ala/Thr tail (CAT tail)^118^. Unlike canonical translation by 80S ribosomes, CAT tailing on the 60S subunit proceeds independently of GTP, suggesting that translational GTPases are not involved^140^. However, the process requires eIF5A, which binds to the E-site of the 60S subunit and facilitates peptidyl transfer^141^.

In yeast, tRNA^Ala(IGC)^ and tRNA^Thr(IGU)^ are selectively recruited by Rqc2 for CAT tailing^118^. In contrast to its selective recognition of incoming aminoacyl-tRNAs, Rqc2 does not discriminate among peptidyl-tRNAs, allowing CAT tailing to initiate on RQC-targeted polypeptides irrespective of their sequence context. During CAT tailing, competition between factors that promote tail elongation (Rqc2 and aminoacyl-tRNAs) and those that mediate peptidyl-tRNA cleavage (Vms1 and Arb1^142^) appears to determine when the process terminates, resulting in variation in CAT tail length. Notably, the amino acid composition of CAT tails differs among species. In *Drosophila*, CAT tails include amino acids beyond alanine and threonine^143^, whereas in humans, they consist predominantly of alanine residues^137,138^. Although CAT tails enhance Ltn1-mediated ubiquitylation (Fig. 4b), they also serve as degradation signals. If stalled polypeptides are released from 60S before Ltn1-dependent ubiquitylation, the CAT tails are recognized by alternative cytosolic E3 ligases, ensuring their clearance^137,144^ (Fig. 4c). In humans, off-ribosome degradation of CAT-tailed proteins may represent an extension of the C-end rule pathway, wherein the identity of the C-terminal amino acid determines protein stability^145,146^. Similarly, the yeast E3 ligase Hul5 has been shown to target a subset of CAT-tailed proteins^144^, although its recognition mechanism may differ from that of the human C-end rule. Notably, non-ubiquitylated CAT-tailed proteins can be released by peptidyl-tRNA hydrolase 1 (Ptrh1) rather than through CCA-end cleavage of the tRNA by ANKZF1^147^. This alternative release mechanism may enhance the efficiency of off-ribosome degradation by more effectively presenting alanine tails (that is, human CAT tails) without conjugated CCA nucleotides to cytosolic E3 ligases^137^.

In yeast, CAT-tailed proteins that escape degradation can accumulate as detergent-insoluble aggregates^7,8^ (Fig. 4d). However, the mechanism by which heterogeneous CAT tails of varying lengths and sequences co-aggregate remained unclear. A recent study demonstrated that threonine-rich polypeptides are particularly aggregation-prone^148^. Surprisingly, polythreonine exhibits a stronger aggregation propensity than polyglutamine^148^, which has been extensively studied for its involvement in human diseases, most notably Huntington’s disease. Among heterogeneous CAT tails, longer tails with higher threonine content can initiate aggregation. These initial aggregates exert a robust seeding effect on other threonine-enriched polypeptides, even when their length is short and threonine content is relatively low, leading to the co-aggregation of heterogeneous CAT tails^148^. In this context, the incorporation of aggregation-prone threonine residues into CAT tails appears counterintuitive if the primary function of CAT tails is to facilitate stalled polypeptide degradation by Ltn1 (Fig. 4b). Sequestering potentially toxic stalled polypeptides into aggregates could serve as a protective mechanism, particularly in rapidly dividing yeast cells^149^ (Fig. 4d). These aggregates may be cleared through autophagy or retained asymmetrically in mother cells to reset proteostasis in daughter cells^150^. However, if CAT-tailed aggregates accumulate beyond a certain threshold, due to aging or persistent stress, they can overwhelm cellular quality control systems and disrupt proteostasis^10^. Therefore, the composition of CAT tails warrants careful examination across organisms, as it may influence the fate and behavior of stalled polypeptides. Notably, mammalian CAT tails composed predominantly of alanine can also promote aggregation, even in the absence or at low abundance of threonine^138,151^.

## Physiological impacts and therapeutic potential of RQC

An increased burden on protein quality control pathways, including RQC, in aged organisms can lead to proteostasis collapse and contribute to age-related protein aggregation disorders. Given its involvement in various diseases, RQC may also represent a potential therapeutic target.

## Disorders associated with impaired RQC

Random mutagenesis using *N*-ethyl-*N*-nitrosourea (ENU) in mice first identified *Ltn1* as a gene associated with a neurodegenerative disorder^12^. The corresponding protein, Listerin, was named after the ‘listing’ (leaning) phenotype observed in mutant mice. Mice carrying a homozygous hypomorphic allele, *lister*, which encodes a protein lacking 14 amino acids, were born without overt abnormalities. However, they progressively developed motor deficits associated with central nervous system (CNS) dysfunction and exhibited a substantially shortened lifespan. Similarly, ENU mutagenesis identified two missense mutations in mouse *Nemf*, R86S and R487G, whose homozygous expression also leads to progressive neuromuscular degeneration^13^. This phenotype is accompanied by atrophy of motor axons—and of sensory axons in the R86S variant—as well as compromised neuromuscular junction integrity. Similar mutations in yeast Rqc2 suggest that the NEMF R86S and R487G variants impair CAT tail extension while preserving core functions such as binding to large ribosomal subunits and recruiting LTN1. These hypomorphic *Nemf* mutations in mice highlight the importance of CAT tailing in maintaining neuromuscular integrity. Notably, NEMF-null mice display an earlier onset and more severe phenotypes than those carrying NEMF missense mutations[13]. Together, findings from *Ltn1* and *Nemf* mutant models underscore the importance of RQC in resolving physiological translation challenges arising from ribosome stalling in vivo. Consistent with these observations, *NEMF* variants have also been associated with juvenile neuromuscular disorders in humans^13,15^. Beyond motor phenotypes, mutations in *NEMF* have been linked to intellectual disability, often accompanied by delayed speech development in early childhood^13,16^. Similarly, *Ltn1*-knockout mice exhibit cognitive impairments^14^. Although neuromuscular deficits were absent in this model—unlike in mice carrying the *lister* allele—these findings reinforce that the CNS is particularly susceptible to impaired RQC activity. Consistently, *Ltn1* knockdown impairs neurite morphogenesis and reduces the viability of primary neurons^138^.

## RQC involvement in *C9orf72*-linked ALS and FTD

In various neurodegenerative disease models, RQC has been identified as a disease modifier, as discussed below. However, it is also noteworthy that severe ribosome stalling can cause neurodegeneration even when RQC function remains intact. In mice, mutation of a CNS-specific tRNA combined with the loss of the putative ribosome rescue factors GTPBP1 or GTPBP2 induces ribosome stalling and, consequently, neurodegeneration^152,153^.

Chromosome 9 open reading frame 72 (*C9orf72*) is the most common genetic cause of amyotrophic lateral sclerosis (ALS) and frontotemporal dementia (FTD)^154,155^. The gene contains a hexanucleotide repeat sequence (GGGGCC, or G4C2) in its first intron^154,155^. In patients with ALS or FTD, pathological expansion of this repeat leads to bidirectional transcription, producing both sense (G4C2) and antisense (G2C4) transcripts^156^. Both transcripts can be translated in all reading frames through a noncanonical mechanism that does not require an AUG start codon^157^. Although the precise molecular details remain to be elucidated, the GC-rich repeat forms stable secondary structures that facilitate this atypical mode of translation initiation, known as repeat-associated non-AUG (RAN) translation^158^. RAN translation of G4C2 and G2C4 transcripts produces five distinct dipeptide repeat proteins: poly(GP), poly(PA), poly(GA), poly(PR) and poly(GR), which accumulate as intracellular aggregates in patients with *C9orf72* expansions^156,157^. Among these, poly(GR) and poly(PR), both containing arginine residues, exhibit the highest cellular toxicity^159,160^. Notably, recent studies have implicated RQC in regulating RAN translation of G4C2 repeats^161–163^. During the synthesis of poly(GR) and poly(PR), the high density of positively charged arginine residues probably induces ribosome stalling through electrostatic interactions within the ribosomal exit tunnel^164^. In addition, the GC-rich G4C2 sequence can form stable RNA secondary structures^165^ that independently trigger ribosome stalling, regardless of the reading frame^163^. Consequently, activation of RQC by overexpressing ZNF598, NEMF, LTN1 or ANKZF1 reduces the levels of RAN translation products, whereas depletion of these RQC factors leads to further accumulation of dipeptide repeats^163^. Interestingly, ZNF598 protein expression, and consequently RQC activity, was found to be reduced in neurons derived from patients with ALS carrying *C9orf72* G4C2 expansions^161^. These findings suggest that genetic or pharmacological enhancement of RQC could help mitigate the toxicity associated with *C9orf72* RAN translation products.

## Mitochondrial dysfunction

Mitochondrial dysfunction is a well-established feature of many neurodegenerative diseases. Notably, such dysfunction has been shown to reduce cytosolic levels of eRF1 and ABCE1, thereby impairing translation termination in human cell lines and *Drosophila*^143^. This cytosolic defect can, in turn, disrupt the mitochondrial proteome, as the vast majority of mitochondrial proteins are nuclear-encoded and translated in the cytosol^166^. Indeed, in *Drosophila* and human cell lines with mitochondrial dysfunction, C-I30, a nuclear-encoded core subunit of complex I in the electron transport chain, undergoes CAT tail extension, indicating ribosome stalling at stop codons caused by impaired cytosolic translation termination^143^. In addition, CAT-tailed C-I30 can be imported into mitochondria and incorporated into the complex I, thereby disrupting respiration and reducing ATP production^143^. Notably, modulation of RQC factor expression, such as repression of NEMF or overexpression of the endonuclease ANKZF1, decreases the production, and probably the proteotoxicity, of CAT-tailed C-I30^143^.

## Cancer

5-Fluorouracil (5-FU) is a widely used chemotherapeutic agent for the treatment of solid tumors. Inside cells, 5-FU is converted into active metabolites that either inhibit thymidylate synthase, an enzyme in nucleotide biosynthesis, or become incorporated into DNA and RNA, thereby exerting cytotoxic effects on cancer cells^167^. Incorporation of 5-FU metabolites into mRNAs can induce ribosome stalling^168^. In addition, 5-FU treatment activates the mTOR signaling pathway, enhancing global mRNA translation and increasing the frequency of ribosome collisions, thereby contributing to its anticancer activity^168^. Notably, *ZNF598*-knockout cells are hypersensitive to 5-FU, potentially due to activation of the RSR^168^. Thus, therapeutic strategies that suppress the RQC pathway could potentiate the efficacy of 5-FU.

## mRNA vaccines

RQC may also influence the efficacy and safety of mRNA vaccines. To increase stability and minimize innate immune response, modified ribonucleosides are commonly incorporated into mRNA vaccines^169^. A recent study showed that *N*^1^-methylpseudouridine, a modification used in severe acute respiratory syndrome coronavirus 2 (SARS-CoV-2) mRNA vaccines, can induce ribosome stalling and subsequent frameshifting^170^. These findings suggest that RQC may help mitigate potential side effects arising from ribosome stalling during translation of mRNA vaccines. Translation of mRNA vaccines generates antigenic proteins, which are processed by the proteasome into peptides for presentation on major histocompatibility complex (MHC) class I molecules. In this context, it is noteworthy that RQC-mediated cotranslational degradation enhances MHC-I antigen presentation^171^ and may thereby improve vaccine efficacy.

## Concluding remarks

Investigations into ribosome stalling-associated quality control mechanisms have led to several unexpected discoveries, including mRNA-independent translation (CAT tailing)^118^, threonine-based protein aggregation motifs^148^ and ribosomal ubiquitylation^63–65^. The emergence of the ribosome collision concept^2^ has further expanded our understanding of the molecular mechanisms underlying the ISR and the RSR^33^. Studies of ribosome stalling have also provided insights into a long-standing question—how SEC61-associated ribosomes are recycled—leading to the discovery of a UFM1-dependent 60S recycling mechanism^132,133^. Although the ribosome has been structurally characterized in great detail, investigations into ribosome stalling and collisions continue to uncover unexpected molecular events and regulatory principles. It is likely that many more unanticipated biological phenomena remain to be discovered in this context. Beyond its mechanistic complexity, ribosome stalling and collisions are increasingly recognized as contributors to neurological disorders, as well as other diseases and aging^172^. Thus, a deeper understanding of ribosome stalling biology holds great promise for advancing molecular medicine.
